# A Simple Method to Locate the Master Knot of Henry Using the Correlation between the Flexor Tendon Length Parameter and the Foot Length

**DOI:** 10.3390/ijerph19042281

**Published:** 2022-02-17

**Authors:** Kwang Rak Park, Won-Jin Park, Si-Wook Lee, Hongtae Kim, Hyunsu Lee, Jae-Ho Lee

**Affiliations:** 1 Department of Anatomy, Keimyung University College of Medicine, Daegu 42601, Korea; airboba@dsmc.or.kr (K.R.P.); wlsk824@naver.com (W.-J.P.); neuroana@dsmc.or.kr (H.L.); 2 Department of Orthopedic Surgery, Dongsan Medical Center, School of Medicine, Keimyung University, Daegu 42601, Korea; shuk@dsmc.or.kr; 3 Department of Anatomy, School of Medicine, Daegu Catholic University, Daegu 42472, Korea; htaekim@cu.ac.kr

**Keywords:** clinical anatomy, flexor hallucis longus, flexor digitorum longus, Henry’s Knot, tendon length, cadaveric study

## Abstract

The precise location of the Master Knot of Henry (MKH) has important clinical significance, but its anatomical definition has not been agreed upon. The purpose of this study is to present a linear regression equation for predicting length variables based on foot length, by evaluating the correlation of length variables related to flexor hallucis longus (FHL) and flexor digitorum longus (FDL), with respect to the location of the MKH. A total of 95 limbs were dissected from 48 adult cadavers, and were fixed in formalin. Measurements were made for the length parameter, with reference to the landmark. The relevance between length variables was analyzed through simple correlation analysis and linear regression analysis. The foot length was 213.69 ± 17.53 mm, MKH-great toe distal phalanx was 140.16 ± 14.69 mm, MKH-FHL insertion was 124.55 ± 13.46 mm, MKH-little toe distal phalanx was 121.79 ± 13.41 mm, MKH-FDL little toe insertion was 109.07 ± 14.16 mm, and the FHL-FDL angle was 33.15 ± 5.39. The correlation coefficient between all the length variables for foot length showed a high positive correlation. We derived a regression equation that can predict the length of each variable. This regression formula is considered to be highly useful because it can estimate the positional relationship of the MKH relatively simply.

## 1. Introduction

The flexor hallucis longus (FHL) and flexor digitorum longus (FDL) are deep groups of muscles in the posterior compartment of the leg. As these muscles descend distally, they become tendons, and the FHL and FDL intersect each other in the mid-portion of the sole. These muscles are finally inserted into the plantar surface of the distal toe [1]. The FDL tendon crosses over the FHL tendon, and this intersection territory is called the “Master Knot of Henry (MKH), equivalently known as “Henry’s Knot” [2]. MKH is commonly used as a synonym for chiasma tendineum plantare (the chiasma plantare (CP)) in most studies. It is considered a very important surgical landmark for clinicians [3,4].

The knot is used as a landmark during the reconstruction procedure, because if the level of tendon harvesting is set based on the location of the MKH, tendon grafting is possible without compromising the function of the toe [5]. For this surgery, various methods for collecting tendon grafts, such as single-incision, double-incision, and minimally invasive techniques, were developed. Depending on the incision method, the access position and the length of the tendon that can be obtained are different.

Although the importance of the precise location of the MKH is emphasized as such, studies about the location of the MKH on the surface are rare, and there is no consensus on anatomical study [6,7]. To our knowledge, no previous studies have been confirmed to predict the length of flexor tendon parameters based on foot length, and to use this to determine the location of the MKH. The purpose of this study is to derive a linear regression equation to predict the length parameters of the FHL and FDL, and to present a method for locating the MKH simply, based on the foot length on the surface. We hypothesized that if a surgeon could simply find the location of the MKH on the surface, with only the length of the foot, it would be useful for effective surgical execution through a minimal incision. 

## 2. Materials and Methods

A total of 95 limbs were dissected from 48 adult cadavers, and were fixed in formalin. Twenty cadavers were female and 28 were male; the cadavers had a mean age of 81.2 (range 51 to 88) years. A detailed dissection was performed by focusing on the FDL and the FHL tendons for all the toes. The soles of the feet of all cadavers were intact when dissected, with no signs of trauma, ulcers, deformities or surgery; these belong to the Department of Anatomy of the Medical School, and were donated for research and educational purposes.

### 2.1. Landmark Setting and Characteristic Evaluation for All Length Variables

**Figure 1:**
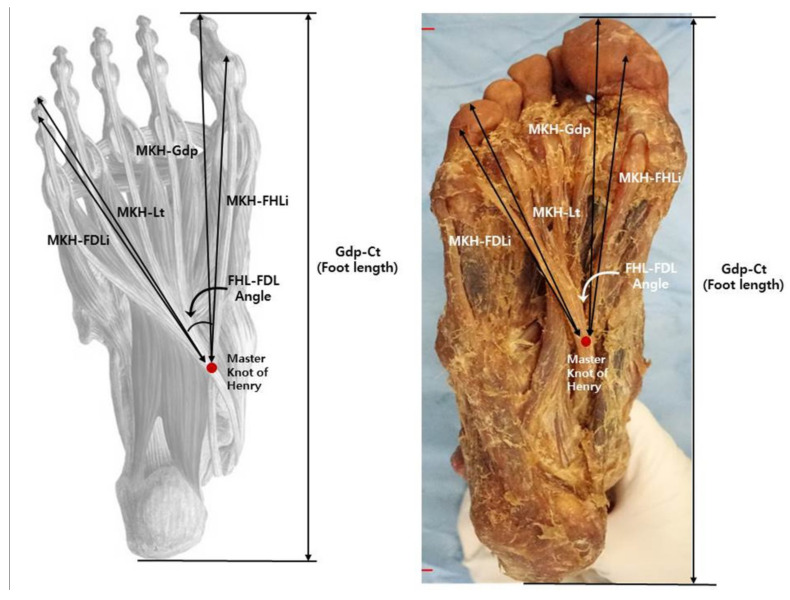
Schematic diagram of the foot showing foot length and MKH-landmark length measurements. Gdp-Ct, great toe distal phalanx-calcaneus tuberosity; MKH-Gdp, Master Knot of Henry-great toe distal phalanx; MKH-FHLi, Master Knot of Henry-FHL insertion; MKH-Lt, Master Knot of Henry-little toe distal phalanx; MKH-FDLi, Master Knot of Henry-FDL little toe insertion; FHL, flexor hallucis longus; FDL, flexor digitorum longus.

The dissection of the sole was performed as a routine procedure from the superficial to the second layer. Landmarks and measurement parameters for length measurement were defined as follows: Gdp-Ct (foot length), from great toe distal phalanx to calcaneus tuberosity; MKH-Gdp, from Master Knot of Henry to great toe distal phalanx; MKH-FHLi, from Master Knot of Henry to FHL insertion; MKH-Lt, from Master Knot of Henry to little toe distal phalanx; MKH-FDLi, from Master Knot of Henry to FDL little toe insertion (Figure 1). MKH was defined as the point where FDL intersects FHL, and the angle between FDL and FHL was measured based on each structure. For all measurements, two main authors agreed on the selection of the measurement point, and the measurements were performed once together. The length of the sole structure was measured with a digital caliper (NA500-300S, Blue bird, Seoul, Korea). Additionally, the angle between the FHL and FDL was measured using a protractor.

The values of all length variables were calculated as percentiles for reference with the foot length: R1, ratio of MKH-Gdp to foot length; R2, ratio of MKH-FHLi to foot length; R3, ratio of MKH-Lt to foot length; R4, ratio of MKH-FDLi to foot length.

### 2.2. Statistical Analysis

All statistical analyses were performed by SPSS (version 24.0, IBM SPSS^®^). Comparison of side and gender for length, and comparison of gender in the ratio of length variable to foot length, were analyzed with an independent sample *t*-test. Correlations among all variables were analyzed by the Pearson correlation test. Scatter plots and linear regression analysis were performed to confirm the relationship between foot length and each length variable. All data were analyzed statistically, and *p*-values less than 0.05 were considered statistically significant.

## 3. Results

For the mean length of the length measurement variable, Gdp-Ct (foot length) was 213.69 ± 17.53 mm (mean ± SD), MKH-Gdp was 140.16 ± 14.69 mm (mean ± SD), MKH-FHLi was 124.55 ± 13.46 mm (mean ± SD), MKH-Lt was 121.79 ± 13.41 mm (mean ± SD), MKH-FDLi was 109.07 ± 14.16 mm (mean ± SD), and the FHL-FDL angle was 33.15 ± 5.39 (mean ± SD). There was no significant difference between all the variables in the length comparison on the left and right sides (Table 1). However, the mean length was found to be longer for men than for women, which is probably a natural result.

The median ratios of the length variables to foot length are 0.66 (range 0.58 to 0.76) for R1, 0.58 (range 0.45 to 0.67) for R2, 0.56 (range 0.48 to 0.64) for R3, and 0.51 (range 0.42 to 0.59) for R4 (Table 2). There was no significant difference in all the ratios between genders (*p* = 0.584 (R1), 0.163 (R2), 0.315 (R3), and 0.721 (R4)).

The correlation analysis results between foot length and the length variables, MKH-Gdp (Pearson r = 0.751, *p* < 0.001), MKH-FHLi (Pearson r = 0.735, *p* < 0.001), MKH-Lt (Pearson r = 0.707, *p* < 0.001), and MKH-FDLi (Pearson r = 0.723, *p* < 0.001), showed a high positive correlation in all the length variables. However, there was no significant correlation between foot length and the angle variables (Table 3).

**Figure 2:**
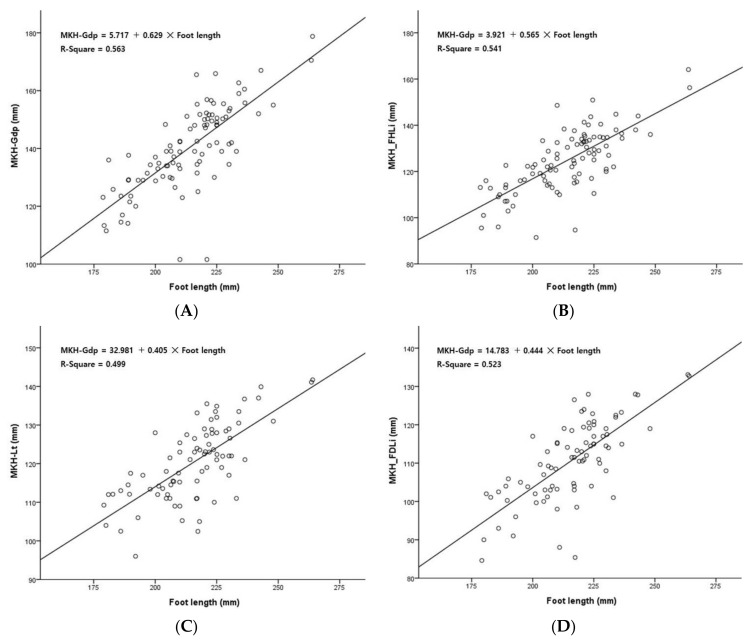
Simple linear regression analysis for prediction of length variables using foot length. (**A**) MKH-Gdp length prediction using foot length; (**B**) MKH-FHLi length prediction using foot length; (**C**) MKH-FDLi length prediction using foot length; (**D**) MKH-FDLi length prediction using foot length.

Figure 2 shows a scatter plot and linear regression line for each length variable versus foot length. As a result of the regression analysis performed to estimate each length variable versus foot length, it was found that foot length had a significant effect on each length variable (*p* < 0.001). The explanatory power of foot length to describe each length variable was 56.3% for MKH-Gdp, 54.1% for MKH-FHLi, 49.9% for MKH-Lt, and 52.3% for MKH-FDLi. Through simple linear regression analysis, the regression equation for the prediction of each length variable, according to foot length, is as follows (Figure 2):

MKH-Gdp = 5.717 + 0.629 × Foot length,(1)

MKH-FHLi = 3.921 + 0.565 × Foot length,(2)

MKH-Lt = 32.981 + 0.405 × Foot length, (3)

MKH-FDLi = 14.783 + 0.444 × Foot length, (4)

## 4. Discussion

We presented the values of all the length variables and the ratio of each length variable to foot length. In the comparison of all the measured lengths, there was no significant difference between the right and left length variables, but in the comparison of genders, men were significantly longer than women in all the length variables (*p* < 0.001). However, in the comparison of the ratio of length variables to foot length, it was found that there was no difference according to gender in all the length variables (*p* = 0.584 (R1), 0.163 (R2), 0.315 (R3), and 0.721 (R4)). In a simple length comparison, it can be assumed that the difference in length between men and women is a result of the difference in foot length, rather than a result of the gender itself. Combining all the results, it would be possible to apply the length variable to the foot length clinically, regardless of the side and gender.

Foot length and the MKH-FHLi length were compared with previous studies. In this study, the foot length (mean ± SD) was 213.7 ± 17.5 mm and the length of MKH-FHLi was 124.6 ± 13.5 mm. In a study of Turkish people [8], the foot length was 232.3 ± 13.0 mm and the length of MKH-FHLi was 126.1 ± 11.1 mm. In a study of Thai people [7], it was suggested that the foot length was 230.9 ± 15.3 mm and the length of MKH-FHLi was 117.1 ± 10.0 mm. In a study of Indian people [9], it was suggested that the foot length was 189.5 ± 30.2 mm and the length of MKH-FHLi was 122.3 ± 10.0 mm. In a Chinese study [10], the length of MKH-FHLi was 10.89 ± 1.08 mm. When the ratio of foot length to MKH-FHLi was calculated, it was found to be 0.58 in our study, 0.54 in the Turkish people, 0.51 in the Thai people, and 0.65 in the Indian people. Various results were confirmed for foot length, MKH-FHLi, and the ratio of MKH-FHLi to foot length. It can be observed that various races have different lengths and proportions.

The MKH is used as an important surgical landmark during tendon transfer for the reconstruction of an Achilles tendon rupture or dysfunction of the posterior tibialis tendon (FHL tendon grafts are obtained using a double incision method, and FDL tendon grafts are obtained using a medial approach) [3,10,11]. Most of the previous studies only suggested the simple distance from the MKH to the reference point for each incision method [7,8]. In this study, a regression equation that can predict the length of MKH-Gdp, MKH-FHLi, MKH-Lt, and MKH-FDLi was presented, and is based on the length of the foot, which can be measured on the surface of the human body. The length of the foot versus MKH-Gdp and the length of the foot versus MKH-Lt on the surface can be used to relatively easily obtain the location information of the MKH before starting the operation. The lengths of MKH-FHLi and MKH-FDLi may be used to determine whether an appropriate length can be obtained as a donor for tendon grafting. 

In this study, in the correlation analysis of the length variables for foot length, the correlation coefficient (r) was 0.751 for MKH-Gdp, 0.735 for MKH-FHLi, 0.707 for MKH-Lt, and 0.723 for MKH-FDLi, indicating high correlation in all the length variables (*p* < 0.001) (Table 3). In a study on American subjects, the correlation coefficient (r) for the length of the Achilles tendon to the length of the foot was 0.491 [12]. Since the correlation of the tendon length variable with the foot length has a greater influence than previous studies on other parts (Achilles tendon), it can be adopted as a useful application method.

In the course of tendon grafting using flexor tendons, the collection of tendon grafts, while setting the level of tendon harvest or preserving toe function, is of great interest to surgeons [5]. Therefore, various surgical methods, such as single-incision, double-incision, and minimally invasive techniques, have been developed. In particular, the double-incision method performed based on the MKH is known as a method used to collect long (about 3 cm) tendons, as opposed to the single-incision method [6,7]. During surgery, the location of the MKH on the surface can be utilized as an important indicator of tendon reconstruction. In this study, a linear regression equation for flexion tendon parameters was derived, in order to confirm the position of the MKH by only measuring the length of the foot. This predictive method can be derived relatively simply, and can be operated through a minimal incision, and can be used to minimize iatrogenic damage.

This study has some limitations. First, we dissected formalin-fixed cadavers. In general, formalin-fixed cadavers may cause deformation of tissues, such as muscles and tendons, but there is no basis for correcting this. Therefore, the procedure of calibration was not followed. Second, for the variable measurements, two main authors selected the measurement point and measured it together, only once. Therefore, its reliability may have some limitations. Third, although previous studies have reported that there may be many types of variant linkages between the FHL and FDL, this study did not present a classification of the linkages between the FHL and FDL. Thus, a follow-up study to supplement this is needed [13,14,15]. Fourth, information on the major nerves and blood vessels passing through the sole could not be provided. Further studies must be performed to understand the relationship between nerves, blood vessels, and various tissues around the MKH of the sole. 

## 5. Conclusions

In conclusion, all the length variables around the location of the MKH had a significant correlation, and the ratio of the length variables to foot length was presented, alongside a linear regression equation of all the length variables to foot length. This is considered to be of high value as basic anatomical data that can predict the length of the tendon or estimate the positional relationship of the MKH relatively simply when a surgeon performs tendon transfer surgery in the clinic.

## Figures and Tables

**Table 1:** Comparison of the left and right sides of the measured structure.

Measurement (mm)	Right (Mean ± SD ^1^)	Left (Mean ± SD ^1^)	Total (Mean ± SD ^1^)	*p*
Gdp-Ct (Foot length)	214.37 ± 18.86	212.43 ± 17.13	213.69 ± 17.53	0.614
MKH-Gdp	140.10 ± 15.88	140.65 ± 14.37	140.16 ± 14.69	0.866
MKH-FHLi	123.83 ± 14.07	125.68 ± 13.58	124.55 ± 13.46	0.529
MKH-Lt	122.09 ± 14.38	122.47 ± 13.06	121.79 ± 13.41	0.903
MKH-FDLi	108.92 ± 15.36	109.65 ± 13.88	109.07 ± 14.16	0.827
FHL-FDL Angle	33.45 ± 6.10	32.69 ± 4.96	33.15 ± 5.39	0.613

^1^ SD = standard deviation. Gdp-Ct, great toe distal phalanx-calcaneus tuberosity; MKH-Gdp, Master Knot of Henry-great toe distal phalanx; MKH-FHLi, Master Knot of Henry-FHL insertion; MKH-Lt, Master Knot of Henry-little toe distal phalanx; MKH-FDLi, Master Knot of Henry-FDL little toe insertion; FHL, flexor hallucis longus; FDL, flexor digitorum longus.

**Table 2:** The ratios calculated based on the measured length values.

Ratio	Median	Range		IQR	
		Min	Max	Q1	Q3
R1	0.66	0.58	0.76	0.63	0.68
R2	0.58	0.45	0.67	0.56	0.60
R3	0.56	0.48	0.64	0.54	0.59
R4	0.51	0.42	0.59	0.50	0.54

R1, ratio of MKH-Gdp to foot length; R2, ratio of MKH-FHLi to foot length; R3, ratio of MKH-Lt to foot length; R4, ratio of MKH-FDLi to foot length; IQR, interquartile ranges; Q1, lower quartile; Q3, upper quartile.

**Table 3:** Correlation of foot length with the length and angle parameters.

	Pearson Correlation (r)	*p*
MKH-Gdp	0.751	<0.001 *
MKH-FHLi	0.735	<0.001 *
MKH-Lt	0.707	<0.001 *
MKH-FDLi	0.723	<0.001 *
Angle	0.226	0.75

*: Significant difference (*p* < 0.001).

## Data Availability

The data used to support the findings of this study are available from the corresponding author upon request.
